# Supplementary material for: Study protocol: LIAM Mc trial (Linking In with Advice and supports for Men impacted by Metastatic cancer)
Source: PLoS One. 2025 Apr 3;20(4):e0313454. doi: 10.1371/journal.pone.0313454 (PMC11967965; doi:10.1371/journal.pone.0313454)
Supplement: S2 File — (PDF) [file pone.0313454.s002.pdf]

The LIAM Mc Trial

STUDY PROTOCOL

Trial Title: The LIAM Mc Trial - Linking In with Advice and supports for Men impacted by Metastatic cancer

Principal Investigator

Professor Jack Gleeson, MB, BCh, BAO, MRCPI

Consultant Medical Oncologist, Cork University Hospital,

Wilton Road, Cork, Ireland.

T.021 4922603

E. mailto:jack.gleeson@hse.ie jgleeson@ucc.ie

Sponsor: University College Cork (UCC) **Sponsor Study Code:** 22052

Principal Project Reference	MHI006	Other project references	ClinicalTrials.gov Identifier: NCT 05946993<br>Cancer Trials Ireland (CTI) number: CTRIAL-IE 23-18
CREC Reference number:	ECM 4 (v) 01/11/2022 & ECM 5 (11) 31/01/2023 & ECM 3 (lll) 09/05/2023		

The LIAM Mc Trial

Protocol Agreement

Title	LIAM Mc Trial - Linking In with Advice and supports for Men impacted by Metastatic cancer
Project Reference	MHI006
Study Sponsor	University College Cork (UCC)
Recruitment Date	June 2023-June 2025
Sites	Mardyke Arena, UCC, Cork and Cork University Hospital, Cork

I, the undersigned, have read and understand the specific Study Protocol, and agree with the contents. The Study Protocol, the Investigator's Agreements and any additional information provided by the sponsor will serve as a basis for cooperation in the study.

I agree to conduct in person or to supervise the study.

I agree to ensure that all that assist me in the conduct of the study have access to the study Protocol plus any amendments and are aware of their obligations.

Site Principal Investigator:

Professor Jack Gleeson	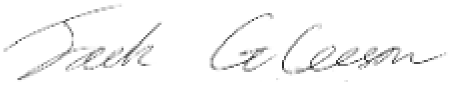	19 July 2023
Name	Signature	Date

Institution	University College Cork/Cork University Hospital

Name and Address and Professional Position of Principal Investigators:

Professor Jack Gleeson, MB, BCh, BAO, MRCPI

Consultant Medical Oncologist, Cork University Hospital,

Wilton Road, Cork, Ireland.

T. 021 4922603

E. mailto:jack.gleeson@hse.ie jgleeson@ucc.ie

Summary of study documents versions

Note: Main Protocol document has its own version and date and each appendix document has its own version and date.

CREC Reference Number: ECM 4 (v) 01/11/2022

CREC amend Ref	Document	Version	Date	Summary of Changes
N/A	Protocol	V1.2	14 Oct 2022	N/A
ECM 4 (v) 01/11/2022<br>& ECM 5 (11) 31/01/2023	Protocol	V1.3	24 Nov 2022	Brendan Noonan – Co-Investigator rather than Co-PI. Addition of supporting appendix documents regarding questionnaires. Adjustment of secondary outcome measures to define questionnaires. Clarification on data collection.
ECM 4 (v) 01/11/2022<br>& ECM 5 (11) 31/01/2023 & ECM 3 (t) 12/09/2023	Protocol	V2	19 July 2023	Change of PI from Dr Bambury to Prof Gleeson. Dr Bambury will now be a Co-Investigator. Contact nurse details changed. Remove Dr Ciara Lyons. Amend recruitment dates for the study to reflect commencement of study. Eligibility criteria edited for clarity. Change wording in dietetics section to reflect change of data collection points and rewording for better clarification of study.
	Protocol	V3	01 Sep 2023	Change of venue from Cardio Rehab Gym, CUH to Mardyke Arena, UCC

<table>
<tr><td colspan="4">Study Synopsis</td></tr>
<tr><td>Brief Title</td><td colspan="3">The LIAM Mc Trial</td></tr>
<tr><td>Title</td><td colspan="3">The LIAM Mc Trial - Linking In with Advice and supports for Men impacted by Metastatic cancer</td></tr>
<tr><td>Principal Project Reference</td><td>MHI006</td><td>Other project reference</td><td>UCC code: 22052
ICS code: MHI22BAM
ClinicalTrials.gov Identifier: 05946993
CTI code: CTRIAL-IE 23-18</td></tr>
<tr><td>Study Design</td><td colspan="3">Feasibility study</td></tr>
<tr><td>Specific Aims</td><td colspan="3">To assess the feasibility of a comprehensive multidisciplinary interventional programme for men living with advanced/metastatic genitourinary cancers</td></tr>
<tr><td>Sampling Method</td><td colspan="3">Sequential Cohorts/ Parallel Sampling Groups</td></tr>
<tr><td>Sample Size</td><td colspan="3">72</td></tr>
<tr><td>Entry Criteria</td><td colspan="3">Synopsis:
Advanced / metastatic genitourinary cancer (including prostate, kidney, urothelial tract, testicular and/or penile cancers), provided they meet the following conditions:
• Currently on active surveillance (i.e. no active systemic therapies at present) or continuing on maintenance systemic therapy, provided they do not have ongoing adverse events which will impact their participation at the time of commencing the 12-week intervention.
• Note: Men with resected disease (adjuvant setting) are eligible if they have commenced or completed adjuvant systemic therapy within the past 12 months and have recovered from these treatments at the time of commencing the 12-week programme from ongoing systemic therapy.</td></tr>
</table>

	• Please see Section 4.1 and 4.2, Inclusion & Exclusion Criteria for full list and description.
Intervention	**Men's Cancer Survivorship Programme:**<br>A 12-week group-based interventional programme is proposed for men with advanced genitourinary cancers, encompassing intensive multidisciplinary input to provide men with personalised tools and coping mechanisms for life with cancer. The programme is based upon providing a supportive and safe enabling environment for the introduction of self-care interventions using a group-based format supported by individualised counselling according to the participant's identified needs. This will involve an allied health professional-led programme to include physiotherapist input twice per week, dietitian input two-weekly, specialist nursing input weekly, medical social worker input and psycho-oncology input weekly, with programme oversight by medical oncologists, in the Mardyke Arena, University College Cork (UCC).
Study Sponsor	University College Cork (UCC)
Recruitment Date	June 2023-June 2025
Primary Endpoint	To evaluate the feasibility of introducing a men's cancer survivorship programme into routine follow up care in patients with advanced genitourinary malignancies.
Dissemination	The results will be considered for submission to an appropriate scientific conference for presentation and/or peer reviewed publication.

**Contacts**	
PI	Professor Jack Gleeson , MB, BCh, BAO, MRCPI<br>Consultant Medical Oncologist, Cork University Hospital,<br>Wilton Road, Cork, Ireland.<br>T. 021 4922603

	E. mailto:jack.gleeson@hse.ie jgleeson@ucc.ie
Co-Investigators	Dr Richard Bambury (Medical Oncologist,CUH)<br>Dr Brendan Noonan (Lecturer Practitioner, School of Nursing & Midwifery, UCC)<br>Ms Anita Cahill (Urology/Uro-oncology candidate Advanced Nurse Practitioner, CUH)<br>Ms Stephanie Corkery (Physiotherapist, CUH)<br><br>Professor Roisin Connolly (Professor of Cancer Research, UCC)<br>Professor Josephine Hegarty (Professor of Nursing, UCC)<br><br>Mr Martin O'Sullivan (PPI Representative)<br>Mr Gerard Ingoldsby (PPI Representative)<br>Dr Mohamad Saab (Nursing Lecturer, UCC)<br>Dr Mike Murphy (Applied Psychology Lecturer, UCC)<br>Ms Katie Johnston (Dietitian, UCC)<br>Dr Samantha Cushen (Dietetics Lecturer, UCC)<br>Dr Fiona Byrne (Dietitian Manager, CUH)<br>Dr Daniel Nuzum (Pastoral Care, CUH)<br>Ms Katarina Medved (Research Assistant, Cancer Research @ UCC)<br>Ms AnneMarie Cusack (Research Nurse, Cancer Research @ UCC)<br>Ms. Aoife Crowe (Pharmacist, CUH)
Statistician	Dr. Darren Dahly<br>HRB CRF-C Senior Lecturer in Biostatistics<br>T. 021 420 5594<br>E. ddahly@ucc.ie

Contact details:	The LIAM Mc Trial,<br>Cancer Research @ UCC,<br>4th Floor, Western Gateway Building,<br>Western Road,<br>Cork.mailto:<br>Anne Marie Cusack CNM 1,<br>Cork University Hospital/University College Cork<br>Phone :0879563385<br>Email: annemarie.cusack@hse.ie/ ACusack@ucc.ie

Table of Contents

1.0 Introduction....11

2.0 Study Hypothesis and Objectives....13

2.1 Hypothesis....13

2.2. Primary Objective:....13

2.3. Secondary Objectives:....13

3.0 Study Plan & Endpoints....14

3.1 Design:....14

3.2 Anticipated Study timeline:....14

Overlapping Cohorts in the Expansion Phase....15

3.3 Primary Study Endpoint:....15

3.4 Secondary Study Endpoints:....16

4.0 Methods....18

4.1 Inclusion Criteria:....18

4.2 Exclusion Criteria:....19

4.3 The 12-week intervention programme....20

4.4 Study procedures....23

4.4.1 Screening....23

4.4.2 Informed Consent....24

4.4.3 Survivorship Programme....24

4.4.4 Instruments for data collection....30

4.4.5 Duration of the Programme....35

4.4.6 Electronic Patient Reported Outcomes (ePRO) Enrollment and Procedures....35

4.4.7 Discontinuation of Subjects....35

4.4.8 Usability and Satisfaction..36

4.4.9 Follow up 37

4.4.10 Data extracted from patient hospital records...37

5.0 Schedule of Activities 38

6.0 Statistical Plan...42

6.1 Sample size justification 42

6.2 Statistical Analysis Plan: 42

7 .0 Safety and Adverse Events..44

7.1 Recording of Adverse Events 44

8.0 Data Handling and Record Keeping...44

8.1 Confidentiality 44

8.2 Source Documentation...44

8.3 Case Report Forms 45

8.4 Records Retention..45

9.0 Ethical Considerations 45

10.0 Study Finances...45

10.1 Funding Source:...45

10.2 Indemnity for the performance of the study..46

11.0 Sponsorship 46

12.0 References...47

13.0 Study Documents Versions Log 49

1.0 Introduction

As highlighted in the National Cancer Strategy 2017-2026 [1], advances in cancer treatment are greatly increasing survival rates for patients. There are now approximately 200,000 people in Ireland living with and beyond cancer. However, the consequences of cancer and its treatment can result in significant, often lifelong, effects on health and quality of life. Patients have many and varied needs on the cancer survivorship trajectory. The health care system will be able to respond to these needs in a more coordinated way if a cancer survivorship pathway is formalised with particular emphasis being placed upon dealing with troublesome symptoms, supporting individuals to transition through the various stages of the cancer journey, encouraging the active participation of patients in care and helping individuals to live well with, through and beyond a cancer diagnosis.

The ICS have funded a number of survivorship research programmes, including the recent Women's Health Initiative [2]. With approximately 13,000 men diagnosed with invasive cancer every year, men in Ireland are more likely to get cancer compared to women, and are 30% more likely to die from their malignancy. Furthermore, at a recent stakeholder workshop held to examine the unmet needs of men affected by cancer in Ireland, contributors highlighted the glaring issues and disparities facing male cancer survivors, and particularly emphasised the challenge of supporting men to engage with cancer survivorship services to derive an optimal outcome for their illness. In line with this, and as part of its strategy [3], Cork University Hospital and University College Cork in association with the Irish Cancer Society (ICS) are dedicated to drive forward this initiative which seeks to improve the experiences, quality of life and outcomes of men impacted by advanced cancer.

"The LIAM Mc Trial – Linking In with Advice and supports for Men impacted by Metastatic cancer" will develop and pilot a dedicated integrated survivorship research programme aimed at better engaging with and supporting men in Ireland post-cancer treatment. As part of this new initiative, we seek to address the key gaps and unmet survivorship needs of men affected by cancer. In particular, an important aspect of this programme is to demonstrate how to improve the survivorship supports and services for underserved communities of men who have not traditionally been the focus of such initiatives and are recognised as experiencing disparities in terms of cancer incidence, prognosis, outcome and/or quality of life. Unfortunately, there are many such communities but these might include, for example, members of the Travelling community, the LGBT+ community, ethnic minority and migrant communities, communities with social disadvantage and/or socio-economic challenges, or specific mental health issues likely to impact their ability to have a positive outcome from a cancer diagnosis. The focus of this programme will be centred on provision of evidence to drive improvement in the survivorship supports and services for those cancers that yield a significant burden on quality of life due to morbidity related to tumour burden, local treatment effects, and/or systemic treatment effects such as androgen deprivation, for which there are still considerable challenges and resources issues.

Male cancer survivors may experience effects of cancer treatment that can have a considerable impact on perceived masculinity through sequalae such as erectile dysfunction and the psychological impact of change of body image, which is further compounded by the lack of supports to express this distress [4]. The LIAM Mc Trial will aim to address some of the most common unmet needs of male cancer survivors (physical, social, and psychosocial) and address their cancer-related symptoms within a holistic and person-centred approach. Recruitment of participants will include a gateway introduction which will allow the men to express any concerns or discuss areas where they require more support. In the Initial Phase, the project team will enrol a cohort of advanced or metastatic prostate cancer survivors as they often experience a significant burden from the side effects of treatment and their advanced cancer status. The larger Expansion Phase will enrol men with any advanced genitourinary malignancy. The programme, which will be underpinned by a robust research infrastructure, will coordinate resources, be readily accessible and seek to develop and improve evidence-based post-treatment services for these men.

The development of a men's Cancer Survivorship programme within the HSE South/South West hospital group is a collaboration between the Irish Cancer Society, Cork University Hospital, UCC Cancer Trials Group (CTG), the Enhancing Cancer Awareness and Survivorship Programmes (ECASP) at the School of Nursing and Midwifery UCC; and regional Cancer Support services including ARC House, Daffodil Centres and Recovery Haven amongst others. This multidisciplinary-led intervention programme will aim to bridge supports provided between the hospital system and community services.

The proposed intervention strategically aligns with patient priorities. Its goal is to identify and manage important symptoms experienced by men impacted by effects of cancer treatment. These are outlined in the National Cancer Strategy 2017-2026.

2.0 Study Hypothesis and Objectives

2.1 Hypothesis

The introduction of a men's malignancy survivorship programme into routine follow up care will be feasible and will result in improved symptom management and quality of life in patients with advanced genitourinary cancers.

Should this intervention prove feasible, we plan to use the data generated from this feasibility study as the basis for a Quality Improvement (QI) initiative for our patients in the future, by offering this programme to all of our patients as a standard component of clinical care.

2.2. Primary Objective:

- To demonstrate the feasibility of introducing a men's cancer survivorship programme into routine follow up care in patients with advanced genitourinary malignancies.

2.3. Secondary Objectives:

- To evaluate the impact of the survivorship programme on quality of life.
- To evaluate the impact of the survivorship programme on cancer related fatigue.
- To evaluate the impact of the physiotherapist and dietetic intervention on body weight, lean body mass, fat mass, muscle strength and nutritional risk over the study period.
- To evaluate the impact of a dietetic intervention by assessing changes in dietary intake and diet quality over the study period.
- To valuate the impact of the survivorship programme on self-care agency and its relationship to quality of life and symptoms experienced.
- To assess the resource utilisation of signposted services and economic impact of a men's cancer survivorship programme.
- To explore and assess the satisfaction of patient and Health Care Professionals with the programme and to determine their perceptions of the systems usability.
- Explore participants' experiences of the intervention focusing on feasibility:
    - the perceived effectiveness of intervention components
    - acceptability of study and intervention procedures
    - the relations between implementation, mechanisms, and context
    - the barriers and facilitators to support effective implementation of future interventional programmes such as this one.

3.0 Study Plan & Endpoints

3.1 Design:

Embedded Mixed-Method Process Evaluation.

This realist process evaluation using a mixed methods design (informed by the Medical Research Council) seeks to address the context of the pilot study, mechanisms of actions, and factors that influence the outcomes of the study i.e. "what works, for whom, under what circumstances" [5,6]. The information gleaned will explain how the intervention worked and how these effects might be replicated in a Quality Improvement (QI) initiative for our patients in the future, by offering this intervention to all of our patients as a standard component of clinical care.

The process evaluation will use the LOGIC model to systematically and visually present, map and share our understanding of the relationships among the resources used to underpin the intervention, the activities planned (intervention inputs, processes, actions) and outputs, outcomes, and impact [5].

Figure 1: Flowchart for LIAM Mc Trial

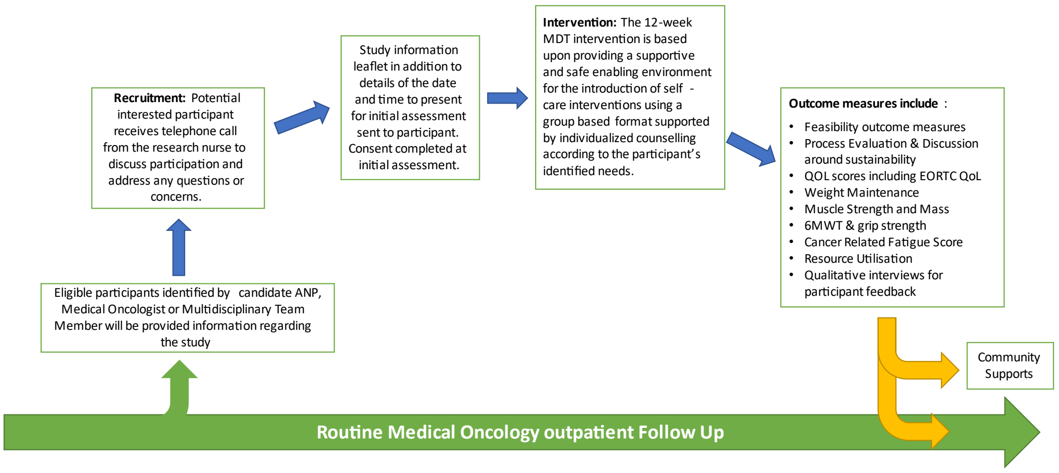

3.2 Anticipated Study timeline:

The study will commence with a scoping review of the published literature to identify clear knowledge gaps and gain an understanding of supportive care interventions previously tested and effectiveness trials/interventions to support men with urological cancer. By conducting a scoping review, the research team will be able to identify the nature of a broad field of evidence in this area to inform the study. This will inform the Initial Phase involving 6 men with advanced prostate cancers. The number of participants is due to Covid-19 health precautions, the availability of the necessary exercise and measurement equipment, and with a view to promoting personalized care plans and to ensure both safety and the best programme possible for these men.

This initial phase, 12-week programme of the first 6 men will be completed and analysed, including follow up and analysis, prior to commencement of the Expansion Phase from Q3 of year 1 onwards, see 3.2.1, Gantt Chart.

In the Expansion Phase, and for the remainder of the study period, parallel sampling groups will be used (i.e. after completion of the 6 participant Initial Phase). A new group of 6 participants will commence the programme every 6 weeks, in order to facilitate workload and resource management, see Figure 2. This will mean two groups are at two different timepoints in the programme at any given date. In total, 12 groups of 6 men are planned, including the initial phase group, with a total anticipated accrual of 72 participants over the two-year study period.

Data collection will occur at three designated timepoints; Baseline/Screening, and End of Treatment visit (after completing the intervention) and at 6 months post intervention. Completion of a process evaluation as part of the feasibility testing and secondary outcome measurement will also occur after all participants have completed the 12-week intervention programme.

Subject recruitment: June 2023-June 2025.

Subject follow up period: September M 2023-l December 2025 (12-18 months recruitment until 72 patients recruited + 6 months follow up for last patient enrolled)

Final data analysis: February 2026-

3.2.1 Overlapping Cohorts in the Expansion Phase

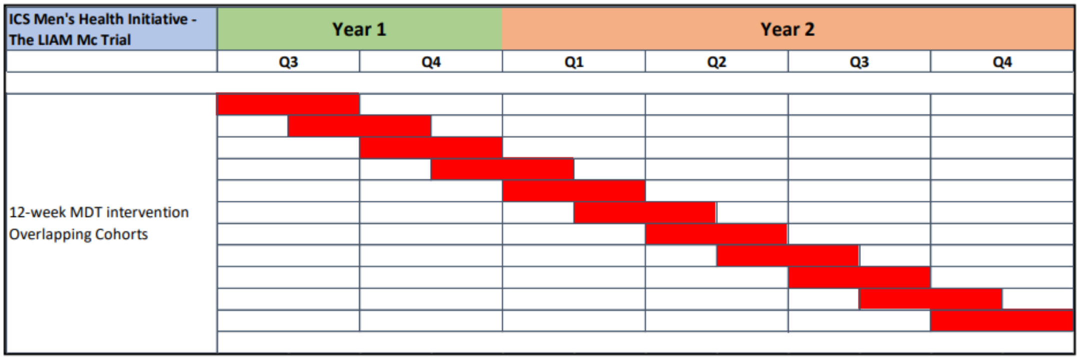

3.3 Primary Study Endpoint:

The primary endpoint of this study is to determine the feasibility and acceptability of introducing a men's cancer survivorship programme into routine follow up care in patients with advanced genitourinary malignancies.

The programme acceptability will be measured with each participant at programme completion and at the 6-month post intervention timepoint. PPI feedback at quarterly engagement meetings will also contribute to understanding the level of acceptability of and support for the study. To determine feasibility of such a multi-disciplinary project, success is multifactorial, and this will be reviewed by the Steering Committee throughout the project and during the scheduled PPI engagement meetings to inform subsequent phases. Qualitative interviews where participant

feedback is provided and the final analysis by the steering and research groups in conjunction with key stakeholders will all contribute to the final determination of the feasibility of the programme. This will help explore what elements of the intervention worked, what elements did not work, and what elements require improvement. A purposefully designed brief feasibility questionnaire will be used in line with previously developed questionnaires by our team [7]. This will be created as part of the scoping review.

3.4 Secondary Study Endpoints:

1. Impact of the survivorship programme on quality of life.
   - EORTC QLQ C30 will be administered at baseline and at the end of the 12-week programme.
   - Quality-adjusted life-year (QALY) (EQ-5D-5L)
   - ASAS-R assessment
   - Additional Assessments as identified by the scoping review will be included in the Expansion Phase.
2. Impact of the survivorship programme on cancer related fatigue.
   - Cancer related fatigue score(EORTC QLQ-FA12).
3. Impact of the programme physiotherapy and dietetic interventions:
   - Maintenance of weight (weight measurement); changes in body composition (lean body mass and fat mass as measured by Bioelectrical Impedance Analysis [BIA]); muscle strength and mass (8 repetition maximum, thigh circumference); physical function and cardiovascular fitness (grip strength, 6 minute walk test [6MWT]).
   - An analysis of body composition analysis using US for muscle mass assessment compared to BIA in a subset of men participating in the study.
   - Changes in dietary intake and diet quality over the study period resulting from the dietetic intervention.
   - Dietary assessments will be conducted by a dietitian. This includes two 24 Hour Dietary Recalls and the WCRF food checklist at baseline and the programme endpoint (12 weeks) and a Food Frequency Questionnaire at baseline and 6 month follow up.
4. Self-care agency and its relationship to quality of life and symptoms experienced.
   - Instruments to measure self-care agency include the ASAS-R
   - Additional Assessments as identified by the scoping review will be included in the Expansion Phase.
5. The resource utilisation of signposted services among participants in the 12-week intervention programme.
   - Quality-adjusted life-year (QALY) (EQ-5D-5L)
   - Resource utilisation (number of signposted resources used). (See section 4.4.4)
6. Satisfaction of participants and Health Care Professionals with the programme and their perceptions of the systems usability.

- Usability, Satisfaction and Feasibility Questionnaire
- Participants, health care professionals and the broader team involved in the development and implementation of the programme will be invited to provide feedback after the completion of their involvement in the study through qualitative interviews.

4.0 Methods

This multi-disciplinary programme will span the hospital system, University and community services. Patients will be recruited predominantly via the Medical Oncology outpatient clinics, see Figure 1, Flowchart. The Patient Information Leaflet (PIL) will be provided to the interested party and upon enrolment the participant will provide Informed Consent.

4.1 Inclusion Criteria:

1. Men aged ≥ 18 years of age at the time of study enrolment.
2. Willing to participate in a 12-week intervention programme and follow up procedures as outlined in the Schedule of Activities section.
3. ECOG performance status 0-2.
4. Recovery to CTCAE Grade ≤2 adverse events from all prior therapies, or adequately recovered adverse events whereby, whereby the PI feels they will not impact the participants ability to complete the 12-week intervention.
5. Disease-specific inclusion criteria:

Prostate cancer:

- High-risk localised (eligible for adjuvant novel anti-androgens), node-positive or metastatic prostate cancer.
- Must have commenced Androgen Deprivation Therapy (ADT) in the form of a gonadotropin-releasing hormone (GnRH) analogue or a GnRH receptor antagonist and/or an androgen receptor antagonist and/or undergone bilateral orchidectomy for prostate cancer. Men must have received last ADT treatment within 12 months of starting the programme or have ongoing treatment-related side effects at the time of commencing the programme if ADT has been discontinued due to toxicity.
- Patients on novel anti-androgen therapy must be stable, and on treatment for at least 3 months, prior to study commencement.
- Patients without histologically confirmed cancer are eligible if both the treating physician and the study PI agree that the patient's history is unambiguously indicative of advanced prostate cancer (e.g. high Prostate-specific antigen (PSA) responsive to ADT in prostate cancer).

Urothelial tract cancer:

- Stage II – IV urothelial tract cancer (muscle-invasive, node positive or metastatic disease) after completion of primary treatment with systemic therapy (including in the neoadjuvant or adjuvant setting) and/or surgery and recovery from these treatments to a satisfactory level.
- Patients with metastatic disease continuing on maintenance systemic therapy are permitted if they do not have ongoing CTCAE Grade >2 adverse events which will impact their participation at the time of commencing the 12-week programme.

Kidney cancer:

- Stage II – III renal cell cancer (clear cell or non-clear cell histologies permitted) after nephrectomy who required and have commenced or completed adjuvant systemic therapy within the past 12 months and have recovered from these treatments to CTCAE Grade ≤2 or do not have ongoing CTCAE Grade >2 adverse events at the time of commencing the 12-week programme from ongoing systemic therapy.
  Or
- Stage IV renal cell cancer (clear cell or non-clear cell histologies permitted) where the participants are continuing on stable dose of maintenance systemic therapy and do not have ongoing CTCAE Grade >2 adverse events at the time of commencing the 12-week programme.

Testicular cancer:

- Stage II-III testicular cancer after completion of primary treatment with systemic therapy and/or surgery within the past 12 months and recovery of all adverse events from these treatments to CTCAE Grade ≤2.

Penile Cancer:

- Stage III – IV penile cancer (node positive, recurrent or metastatic disease) after completion of primary treatment with systemic therapy (including in the neoadjuvant or adjuvant setting) and/or surgery and recovery from these treatments to CTCAE Grade ≤2.
- Patients with metastatic disease continuing maintenance systemic therapy are permitted if they do not have ongoing CTCAE Grade >2 adverse events which will impact their participation at the time of commencing the 12-week programme.

6. Participation in other translational or interventional clinical trials is permitted provided the above disease-specific inclusion criteria are met.
7. Signed consent form by the participant or a legally authorized representative (LAR).

4.2 Exclusion Criteria:

1. Persons who, in the opinion of the researcher or supervising clinician, are unable to cooperate adequately with the study protocol, for example those receiving systemic therapy for a concurrent cancer diagnosis, those with organ system dysfunction which would impact their safe participation in the study, or other uncontrolled medical illness that would impact their safe participation in the study.
2. Patients commencing (in the first 3 months of active systemic therapy) which will impact their ability to participate in the programme are not eligible for the study. These patients may be re-assessed for eligibility when they have completed their therapy or are stable on therapy for at least 3 months.
3. Recent (within 12 months) participation in a study/programme involving a lifestyle intervention (e.g diet, exercise, survivorship).
   a. Note: Per discretion of PI as to whether may impact the outcome of this study intervention.

4.3 The 12-week intervention programme

The programme will involve twice-weekly visits from week 1 – 12 (1 x 1.5 hour session, 1 x 1 hour session). Visits will take place at the Cardiac Rehabilitation Centre in CUH (ARC House, the Mardyke Arena, University College Cork (UCC) or other venues may be used in the event of logistical changes as the study expands from Initial to Expansion Phases).

4.3.1 Screening

A 6-week screening period, to assess eligibility and capture baseline assessments prior to commencing the 12-week intervention programme will involve a screening visit and time for consent. At the Screening visit(s), baseline assessments will include the following:

1. Baseline questionnaire/needs, burden of disease and quality of life assessment
2. Dietetic Evaluation
3. Physiotherapy Evaluation

Information on the questionnaires, evaluations and consent processes are contained in the relevant sections below.

Participants who exceed the 6-week screening period before commencing the 12-week intervention programme may be re-screened for eligibility in a later group. Baseline measurements may need to be repeated if this is the case.

4.3.2 The 12-week intervention programme

The 12-week intervention programme will include twice-weekly exercise & assessment sessions for 1-1.5 hour(s). In person visits will be considered optimal, to aid group discussion and peer support, but virtual participation options will be explored as the trial progresses.

The programme referred to here is a guide – sessions may be reordered depending on healthcare professional availability or other factors, as required.

12-week Intervention Programme – Provisional Schedule

Twice weekly visits from week 1 – 12 (1 x 1.5 hour session, 1 x 1 hour session)

**Week 1**	Introduction to programme<br>Individual Baseline assessments (including Nutritional, Physio and Nursing assessments, burden of disease and quality of life experience)<br>Introduction to signposting to other relevant services
**Week 2**	Nutrition assessment/education (comprehensive diet history, anthropometric measurements, dietary analysis, individualised dietary advice)
**Week 3**	Social work education session<br>(Stress management & Psychosocial support)<br>Nurse present to discuss symptoms
**Week 4**	Nutrition assessment/education (revisit dietary analysis results and adjust tailored advice as appropriate)
**Week 5**	Occupational Therapy (sleep hygiene)<br>Nurse present to discuss symptom management.
**Week 6**	Physiotherapy (Fatigue management, pacing strategies, task prioritisation)<br>Reminder of other available relevant services (Signposting)
**Week 7**	Nurse led Education session on symptom management of side effects of treatment
**Week 8**	Nutrition assessment/education (revisit dietary analysis results and adjust tailored advice as appropriate)
**Week 9**	Pastoral Care<br>(Spirituality & Emotional reasoning)
**Week 10**	Nutrition assessment and education (revisit dietary analysis results and adjust tailored advice as appropriate).
**Week 11**	Final Session to signpost available support services<br>Nurse present to discuss symptom management
**Week 12**	Final measurements<br>Patient feedback questionnaires and thank you session
**After**	Invitation to Quarterly PPI engagement meetings & PPI Workshops to provide feedback for service improvement
**Month 6**	6-month follow-up assessment

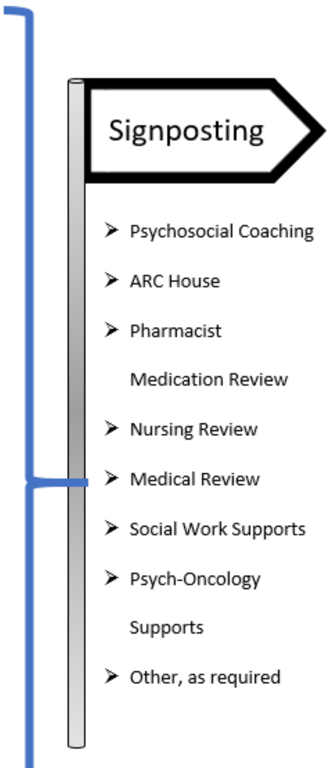

Note: These services will be available at all times, with particular signposting and reminder sessions as highlighted in the schedule.

* Weeks 1-12 will include twice weekly Physio exercise sessions for 1hr

** Visits will take place at the Cardiac Renal Centre Gym in CUH initially, however we are exploring the options of ARC House, the Mardyke Arena, University College Cork (UCC) or other venues for expansion in later phases (see Letters of support).

*** In person visits will be considered optimal, to aid group discussion and peer support, but virtual participation options will be explored as the trial progresses.

**** Above programme is a guide – Sessions may be reordered depending on healthcare professional availability or other factors, as required.

A series of educational sessions will be incorporated in the programme on a weekly basis, including, but not limited to:

1. Nurse-led education session on symptom management of side effects of treatment
2. Social work education session on stress management & psychosocial support
3. Pastoral care session on spirituality & emotional reasoning,
4. Combined occupational therapy / physiotherapy session on fatigue management, pacing strategies, task prioritisation and sleep hygiene

In addition, regular activities will include:

1. Twice-weekly physiotherapy sessions & plan adjustment
2. 2-weekly dietetics assessment & plan adjustment
3. 2-weekly nursing and psychosocial reviews
4. Signposting to additional available resources including but not limited to
   a. Psychosocial Coaching
   b. ARC House
   c. Pharmacist Medication Review
   d. Nursing Review
   e. Medical Review
   f. Social Work Supports
   g. Psycho-Oncology Supports
5. Optional weekly psychological coaching in a virtual format via UCC

The order of the planned activities may be switched between weeks (e.g. week 3 and week 4 on the Study Calendar could be swapped due to HCP availability but all 12 weeks of planned activities will happen).

Symptom pathways will be available for common symptoms identified such as joint pain, depression, anxiety, hot flashes, cognitive impairment and fatigue.

Patients will undertake assessments at baseline, 12 weeks and 6 months. Triggers to the study team will prompt symptom evaluation and management in between routine clinic visits. A phone call will take place at 6 months to follow up on progress, symptoms etc.

The candidate ANP will work alongside staff at the Mardyke Arena, UCC; supervised by consultants and supported by a research assistant. This latter position will involve development of educational materials, promotional material, educational events, data collection etc. A research nurse will coordinate research data collection and analysis under the supervision of Prof Gleeson and the wider research team.

4.3.3 End of Programme/Treatment Visit

At the end of programme/end of treatment visit, follow up questionnaires, burden of disease, nutritional, exercise and quality of life assessments will take place. This can be completed in the

final week of the intervention or within 14 days of programme completion. Full details and further information on the questionnaires, evaluations and processes are contained in the relevant sections below.

4.4 Study procedures

Research and relevant Clinical Staff will be trained in the protocol. Eligible patients identified from routine medical oncology follow up care will be identified and offered participation in the study, as per the screening procedures below. Willing participants will then complete the Ethics Committee approved informed consent process, after which a unique study identifier will be applied to all subject's study data and will be used exclusively as a unique identifier in study Case Report Forms. Patients will then commence the 12-week intervention programme, as outlined below, with data capture and storage at the pre-specified timepoints. A 6 month follow up phone call and completion of questionnaires will also be scheduled. Patients who participate in the 12-week intervention programme will be invited to provide feedback individually on the programme and at the quarterly PPI engagement meetings. Some participants will also be invited to one-to-one or focus group qualitative interview feedback sessions if they consented to participate in these at the outset of the programme. All data captured will be pseudo-anonymised and stored on the electronic CASTOR database in line with UCC's data management policies.

4.4.1 Patient Identification

All patients in routine medical oncology follow up care will be eligible for screening for the study. Patients will be approached and encouraged to self-refer to the programme by a named study investigator. Candidates for the programme will be identified via Uro-oncology multi-disciplinary meetings, the medical, surgical and radiation oncology clinics and direct from our Clinical Nurse Specialists, candidate ANP, consultants and GP practices. We anticipate the majority of patients will be enrolled at a clinic visit with their medical oncologist. Men from traditionally underserved communities who have not typically been the focus of such initiatives and are recognised as experiencing disparities in terms of cancer incidence, prognosis, outcome and/or quality of life will be actively recruited to the study to better understand the impact of this type of intervention on men in these groups. Unfortunately, there are many such communities but these might include, for example, members of the Travelling community, the LGBT+ community, ethnic minority and migrant communities, communities with social disadvantage and/or socio-economic challenges, or specific mental health issues likely to impact their ability to have a positive outcome from a cancer diagnosis.

4.4.2 Informed Consent

Patients will be consented by a delegated member of the study team using the most recent version of the LIAM Mc study Participant information leaflet (PIL) and Informed Consent Form (ICF). This may occur in person, as they present for their routine clinic visit or at the initial programme visit. The completed ICF (signed and dated by both participant and research team member) will be filed in the study file which is kept securely. A copy will be given, emailed or posted back to the participant for their own records.

Alternatively, a remote consenting process (e.g. pending geographic or COVID-related barriers) may be employed, as follows:

- The potential participant will receive a hard copy of the PIL/ICF by email or post.
- A member of the study team will telephone (or may use skype, zoom etc.) the potential participant, and go through the PIL and answer any questions which participant may have
- If the patient is happy to take part the researcher will ask them to sign and date the ICF and send it back to the study team by email (scan the document or take photograph and send as email attachment) or by post.
- The member of the study who explained the study to the patient will then sign and date the ICF.
- The completed ICF (now signed and dated by both participant and research team member) will be filed in the study file which is kept securely. One copy will be placed in the patient's medical notes and one copy will be emailed or posted back to the participant for their own records.
- Once the signed ICF has been filed in the study file it will be deleted from email system.

4.4.3 Survivorship Programme Procedures

The survivorship programme is a 12-week group-based interventional programme for men with advanced cancer, encompassing intensive multidisciplinary input to provide men with personalised tools and coping mechanisms for life with cancer. The programme is based upon providing a supportive and safe enabling environment for the introduction of self-care interventions using a group-based format supported by individualised counselling according to the participant's identified needs. This will involve an allied health professional-led programme to include physiotherapist input twice per week, dietitian input every 2 weeks, specialist nursing input every 2 weeks, medical social worker input and psycho-oncology input, with programme oversight by medical oncologists, in the Mardyke Arena, UCC. An outline of the programme is provided in the 12-week intervention programme (Section 4.3.2) and Study Calendar (Section 5.0).

Research and relevant Clinical Staff will be trained in the protocol. Following successful completion of the Ethics Committee approved informed consent process, a unique study identifier will be applied to all subject's study data

and will be used exclusively as a unique identifier in study Case Report Forms. Subject's clinical notes will be reviewed, and an appointment arranged for the baseline visit.

The initial visit in the Mardyke Arena, UCC will assess and manage current symptomatology and needs per management pathways, refer to appropriate specialists, and ensure future access to the clinic where needed during the study period. Patients will receive education, exercise, diet and symptom management plan, depending on the outcome of the baseline assessments, or according to clinical judgement. The baseline physical and nutritional assessments will be undertaken at this visit. Diet education and personalised nutrition counselling will be performed throughout the study (For further information see Nutritional Intervention section 4.4.3.2).

A study evaluation will take place at the evaluation stage of the Initial Phase, of potential barriers that participants perceive would prelude them in attending face to face. We anticipate it could be travel or access to translator or family commitments. It is the intention of the group to look at a feasible means to address these barriers for later phase participants. If participants cannot attend the scheduled sessions on a given day then virtual meeting or recording options will be explored so that they can still receive the education session.

4.4.3.1 Physiotherapy

Each participant will have 2 x 1h exercise classes per week with a physiotherapist, who will develop individualized plans based on medical history and scan reviews. Participants will be progressively guided through the programme, incrementally increasing in intensity or with modifications based on symptomatic presentation. This will be based on their baseline strength and cardiovascular fitness testing and grounded on evidence-based protocols previously demonstrating an effect in this patient population. Individualised tailored exercise programme which includes strength and conditioning.

Physiotherapy Outcome Measures Assessment Schedule:

Baseline body measurements included will include weight, waist/hip circumference, BMI, Body Composition, muscle strength and cardiovascular fitness (see Section 4.4.4.1 for additional details) and will be measured at the screening visit.

Outcome measures at 12 weeks +/- 14 days (end of programme visit) and a 6 months follow up phone call to assess current physical activity levels will be compared to baseline assessments.

Safety and tolerability Assessment:

Medical clearance will be established, ECOG performance will be evaluated.

Review of previous imaging assessing the location, type of, history and progression of bone metastases will be defined at the outset.

Bone pain details at rest, ADL's and with physical activity will be recorded.

Incidence and severity of any adverse event and skeletal complications related to and unrelated to exercise will be recorded throughout the programme.

Physical function:

A series of standardised tests will be used to test the following physical function:

6 or 8 multiple repetition maximum test will be avoided or use caution if it placed stress on a lesion site for example exclusion of leg press for participant with proximal femur fracture it would be replaced with a 8 RM leg extension test or rib/thoracic metastases chest press would be replaced with a bicep curl test.

The exercise intervention:

Participants will complete twice-weekly combined resistance and high/moderate intensity aerobic sessions.

It will be undertaken in small groups of up to 6 participants for approximately 60 minutes under the supervision of a physiotherapist, dietitian and candidate Advance Nurse Practitioner in an exercise clinical setting.

Exercises will target the major muscle groups

The exercises will be performed based on a modular approach to avoid loading the affected area and minimising the mechanical forces placed on the affected structures, based on the table below.

	Resistance			Aerobic		Flexibility
**Metastases site**	**Upper**	**Trunk**	**Lower**	**WB**	**NWB**	**Static**
Pelvis	√	√	√**		√	√
Lumbar spine	√		√		√	√***
Thoracic spine/ribs	√*		√	√	√	√***
Proximal femur	√	√	√**		√	√
All regions	√*		√**		√	√***

√, target exercise region; *, exclusion of shoulder flexion/extension/abduction/adduction – inclusion of elbow flexion/extension; **, exclusion of hip extension/flexion – inclusion of knee extension/flexion; ***, exclusion of spine/flexion/extension/rotation; NWB, nonweight bearing (e.g. cycling); WB, weight bearing (e.g. walking). Reproduced from [102].

https://www.researchgate.net/profile/Nicolas-Hart/publication/317013384/figure/tbl1/AS:670328686641169@1536830221355/Modular-multimodal-exercise-programme-for-patients-with-bone-metastases.png

Exercise will be progressed from a 12 to 8/6 RM with 2-4 sets and auto-regulated (i.e. the patient progresses at their own pace based on variations in health, performance capability, scheduling commitments, etc. with adjustments made each session according to the patients capacity on the day of exercise)

This allows the patient/therapist to lower the intensity if the patient is fatigued/unwell or raise volume/intensity if the patient is motivated/energised.

Resistance training will not be performed to neuromuscular failure but rather ceased with 1-2 repetitions short of failure of a participant being unable to complete a repetition.

RPE SCALE BASED ON REPETITIONS IN RESERVE	
10	Could not do more reps or load
9.5	Could not do more reps, could do slightly more load
9	Could do 1 more repetition
8.5	Could definitely do 1 more repetition, chance at 2
8	Could do 2 more repetitions
7.5	Could definitely do more 2 more repetitions, chance at 3
7	Could do 3 more repetitions
5-6	Could do 4 to 6 more repetitions
1-4	Very light to light effort

Aerobic exercise intensity is prescribed using the RPE method. Aerobic speed/intensity will be adjusted to elicit the target RPE throughout the programme.

In addition to the clinical setting the participants will be encouraged to complete prescribed home-based exercise sessions of moderate intensity.

RPE Scale	Rate of Perceived Exertion
10	**Max Effort Activity** Feels almost impossible to keep going. Completely out of breath, unable to talk. Cannot maintain for more than a very short time.
9	**Very Hard Activity** Very difficult to maintain exercise intensity. Can barely breath and speak only a few words
7-8	**Vigorous Activity** Borderline uncomfortable. Short of breath, can speak a sentence.
4-6	**Moderate Activity** Breathing heavily, can hold short conversation. Still somewhat comfortable, but becoming noticeably more challenging.
2-3	**Light Activity** Feels like you can maintain for hours. Easy to breathe and carry a conversation
1	**Very Light Activity** Hardly any exertion, but more than sleeping, watching TV, etc

4.4.3.2 Dietetics

This will include both a generalised and tailored approach to maintaining the optimal healthy diet to promote wellness in cancer survivorship.

Each man will receive dietary assessment and counselling two-weekly. The dietitian will modify the dietary plan as required for each man as he moves through the program and treatment.

Patients will complete a symptom survey at intervals throughout the study. The symptom survey using the malnutrition screening tool (MST) will act as a screening tool to identify nutritional risk. Patient-specific nutrition counselling will be offered to patients identified at risk.

Nutrition Education and Personalised Nutrition Counselling:

The main goal of the nutritional intervention is to improve the diet quality of each patient using a standardized nutrition assessment, offering evidence-based group education and developing personalized nutrition goals. Participants will be progressively guided through the nutrition education programme; with modifications to the individual’s usual diet, thereby recognizing personal eating patterns and preferences, which form the basis for individualized dietary counselling. The dietary counselling identifies the type, amount, and frequency of feeding and specifies the caloric/protein level to attain, together with any restrictions and limited or increased individual dietary components. In addition, nutritional advice can be adapted to respond to specific nutrition impact on symptoms (e.g. dysphagia, constipation, diarrhoea, altered taste and temperature sensitivity). Energy and protein requirements will be calculated only for patients that require specific caloric and protein targets to meet their individualised nutritional goals.

Throughout the nutrition education programme, every patient will be educated on diet and lifestyle factors associated with cancer prevention, based on the World Cancer Research Fund's (WCRF) recommendations [8]. After the initial nutrition assessment, dietetic follow up will consist of group dietary counselling 2weekly. Participants may require additional individualised dietary counselling sessions, at the dietitian's discretion based on clinical judgement.

4.4.3.3 Nursing & Psychosocial

This will consist of generalised discussions on areas such as erectile dysfunction, changes in masculinity, effects of hormone treatments on mood changes and body image. There will be an inclusion of practical information sessions including health systems information and managing side effects such as urinary symptoms, hot flushes, penile shortening, and loss of body hair. There will be opportunity for each man to discuss intimate concerns in a quiet and safe space if they so wish.

Nursing, Psycho-oncology, pastoral care and social work-led sessions to inform participants on items including diagnosis shock, acceptance, coping with relationship changes with their partner and their roles, fear of uncertainty and the future, medication management, masculinity (male values being strong, capable, independent), practical management of bills, and household tasks.

Dr. Mike Murphy, in conjunction with the Psychology Dept in UCC, will offer pro bono psychological coaching to all participants in this study individually, to be conducted by supervised MSc students virtually for the men at a time that is convenient.

4.4.3.4 Signposting

Throughout the programme, signposting of additional relevant services will also be conducted, with particular emphasis placed on these at assigned times in the schedule. This is in addition to the group sessions from key HCP's from the social work, pastoral and nursing teams as outlined in the LIAM Mc programme of scheduled activities (Section 4.3.2).

At the baseline clinical visit and as detailed in the Study Calendar, patient will be advised to use publicly accessible resources currently used in standard clinical care including, but not limited, to the following:

a. Psychosocial Coaching
b. ARC House
c. Pharmacist Medication Review
d. Nursing Review
e. Medical Review
f. Social Work Supports
g. Psycho-Oncology Supports

4.4.4 Instruments for data collection

Dose and Reach

The time taken to deliver the intervention will be collected (dose). Level of engagement with the interventions will be scored on a Likert scale (one item on the usability scale). Reach relates to the proportion of the intended population participating, demographic details of participants will be collected and shared anonymously. Targeted recruitment will be used to encourage participation of a demographically heterogenous sample.

Survey Questions

The survey will include open-ended questions asking if the participant had any comments about their experience of participating in the intervention/study; elements of the intervention that worked/didn't work and changes they would like to make.

Other process evaluation data will be collected during the study e.g. timing and difficulties experienced with program components, frequency of adverse events (if any), implementation problems. Implementation problems identified during the study will be catalogued as they arise including the mechanisms by which issues are addressed.

Participants will be asked to complete a brief feasibility questionnaire adapted from Zellinger et al (2015) and based on the work of Dr Saab and Prof Hegarty (both co-applicants) [7], to assess the applicability, acceptability, practicality and relevance of the intervention. The survey will include Likert scale items relating to satisfaction with intervention components.

Qualitative semi structured individual and focus group interviews involving intervention participants and HCPs working within the broader interdisciplinary team will be conducted. The research team including the interventionalists, PPI representatives and key stakeholders (e.g. NCCP representative Dr Terry Hanen) will be asked to partake in individual interviews to glean the perspectives of those delivering the intervention and the wider context into which the intervention is delivered.

A semi-structured interview guide will guide an open, in-depth interview with participants about their experience of the trial/intervention, how participants made sense of the intervention, the acceptability of the interventions, their engagement in the processes of the interventions, and the barriers and facilitators encountered.

In addition the interviewer will explore the mechanisms through which the intervention brings about changes which is crucial to understanding both how the effects of the specific intervention occurred and how these effects might be replicated by similar interventions in the future (MRC, 2015).

Context includes anything peripheral to the intervention that may influence its implementation, reach, or its effects. Thus, understanding the context is critical to understanding the potential impact of context on the implementation of the intervention.

In addition to assessing the overall feasibility, each secondary outcome measure will be measured using reliable and validated tools. The effect of the intervention on key datapoints will be surveyed including physical, diet and psychological measurements, patient reported outcomes, i.e. health related quality of life (HRQOL) and symptoms experienced. Generally, HRQOL covers the subjective perceptions of the positive and negative aspects of cancer patients' symptoms, including physical, emotional, social, and cognitive functions and, importantly, disease symptoms and side effects of treatment.

Data related to patient comorbidities, medication, and clinician record of actions/follow-up at survivorship and symptom management encounters:

Data relating to resource utilisation will be collected on an ongoing basis for the study group. Data relating to survivorship services that were provided by CUH or UCC and used during the study period will be recorded. Data on service utilisation (outside of CUH) will be collected throughout the study period by the information provided from the patient through the study patient's reports at each session and the 6-month follow up; this will include the services used, frequency of use, duration of each service and the personnel responsible for providing the service. This information will be collected by the Research Nurse.

4.4.4.1 Nutritional & Anthropometric assessments

Participants will undergo a baseline assessment during the screening visit or prior to Week 1 and at Week 12, as per the Schedule of Activities, Section 5.1. The following Nutritional & Anthropometric measurements will be completed:

Anthropometric measures:

- **Body weight:** Weight will be recorded with the participant wearing light clothes and no shoes on the same scale at the baseline visit and at the programme endpoint (12 weeks). Patients will also self-assess their weight using the same personal scales at home, at the same time of the day, at baseline and at the endpoint of the study. Participants will report their weight on the same day their weight has been recorded at home through their regular ePRO surveys.
- **Height:** The participant's height will be measured at baseline.
- **Body Mass Index (BMI):** This will be calculated using the participant's weight and height and is part of the WCRF/AICR scoring system.
- **Body Composition:** Segmental body composition will be analysed using Bioelectrical Impedence Analysis (BIA) with the Body Composition Monitor (BCM) (Fresenius Medical Care, Bad Homburg, Germany) for all participants. This is used for an accurate and objective assessment of overall body composition and hydration status [9]. The BCM device calculates the lean tissue mass (LTM), lean tissue index (LTI), fat tissue index

(FTI), body cell mass (BCM), and adipose tissue mass (ATM), extracellular (ECW), intracellular (ICW) and total body water (TBW) based on measurement values, using physiological modelling and mixture equations (Cole-Cole plot and Hanai formulae and Chamney et al, 2007 [10]). Body composition using BIA will be assessed at baseline and the intervention completion (12 week) timepoints. Some patients may also have body composition analysis using a handheld ultrasound Vscan Air™ (GE HealthCare, 2023), for muscle mass assessment by a an appropriately trained member of the study intervention team.

- **Waist circumference:** This measurement will be recorded with the participant not wearing clothing that is directly over the skin. If this is not possible, the measurement may be taken over light clothing. This is also part of the WCRF/AICR standardised scoring system.
- **Muscle strength:** Hand grip strength (HGS) will be measured using the Jamar dynamometer (Jamar Hydraulic Hand Dynamometer, Model 091011725, Sammons Preston Roylan, Nottinghamshire, UK) with its handle in the second position as recommended by the American Society of Hand Therapists (ASHT) [11]. Grip strength will be measured 3 times per hand, alternating between left and right hand to allow rest and account for any fatigue.
- **Nutritional risk:** Participant's nutritional status will be monitored over the 12-week period on the study database by assessing any change in their Body Composition and MST score, see below.

Dietary quality will be measured using the WCRF/AICR standardised scoring system which was discussed earlier. This also includes assessment of their alcohol intake.

Dietary intake: Dietary assessments will be conducted by a dietitian. This includes two 24 Hour Dietary Recalls and a Food Frequency Questionnaire at baseline and month 6 (questionnaire follow up). Portion sizes will be validated through the use of a food atlas.

Additional detail on instruments for data collection of the Nutritional & Anthropometric assessments are provided here:

- **24 Hour Dietary Recall:** Following assessment of the 24h diet recalls, the programme dietitian will assign appropriate food codes to each item reported. Data analysis will be limited to the McCance and Widdowson's composition of foods integrated dataset, the USDA Food Composition Databases and the Irish food composition databases. Food coding will be completed following each individual dietetic assessment. A food code data base will be generated and expanded upon following extraction of new food codes from each new 24hour diet recall recorded. This data will be analysed using Nutritics, web-based nutritional analysis software to capture diet intake assessing macro- and micronutrients, and specific food and nutrients directly relating to the dietary quality outlined in the WCRF framework for cancer prevention. All reference data will be assessed within-individual comparison and between-individual comparison. All data will be exported from Nutritics into a Microsoft excel file for processing with the study statistician.

- **Food Frequency Questionnaire:** Individual and collective sample data captured from the EPIC-Norfolk Food Frequency Question (FFQ) will be entered in to the FFQ processing tool, FETA (Φετα) FFQ EPIC Tool for Analysis, which is based on the earlier CAFÉ system [12]. The FETA tool is free to use and will produce different levels of nutrient data, as well as basic food groups and food patterns. This programme is based on version 6 (CAMB/PQ/6/1205) of the EPIC-Norfolk Food Frequency Questionnaire (FFQ). FETA calculates the average daily intake of 46 nutrients and 14 food groups, for each individual. The default nutrients list provides a description of each nutrient/food group and the units used. The nutrient data for the FFQ foods have come from McCance and Widdowson's "The Composition of Foods (5th edition)" and its associated supplements. In FETA, the frequency category is converted into a portion multiplier (e.g. once a week = 1/7 = 0.14). After multiplication with the portion size, an average daily food weight for each of the 130 FFQ items is obtained. These weights are multiplied by the nutrient composition per gram to obtain the nutrient composition of the actual amount eaten. After summing all FFQ items for a participant, an average daily nutrient intake is obtained. Individuals with more than 10 missing lines of data will be excluded. The top and bottom 0.5% of the ratio of energy intake to estimated basal metabolic rate will be flagged as extreme outliers of nutrient intakes. All data will be exported from FETA data processing report into a readable Microsoft excel file for processing by the study statistician.
- **Malnutrition Screening Tool:** The MST is a simple, quick, valid and reliable tool which can be used to identify patients at risk of malnutrition. It asks participants if they have lost weight recently without trying (and if so, how much) and if they have been eating poorly because of a decreased appetite [13]. Patients identified at nutritional risk, will be advised on an appropriate nutrition care plan. Some patients may require ongoing monitoring, nutritional counselling and review of personal nutrition care plans. These patients will be invited to attend the ongoing dietitian clinic. The need for follow up review and number of sessions required for each patient will be decided by the programme dietitian based on clinical judgment.
- **Physical activity:** a short validated physical activity questionnaire will be used [14] to measure physical activity levels. Physical activity is a fundamental Cancer Prevention Recommendation of the World Cancer Research Fund/American Institute for Cancer Research (WCRF/AICR) framework for cancer prevention. It will be measured as moderate to vigorous physical activity in minutes per week.The data for physical activity will be collected by the physiotherapist, which the dietitian will extract and place in a scoring system. It will be included in the (WCRF/AICR) standardised scoring system [8]. This is a practical tool that operationalises eight out of ten 2018 WCRF/AICR cancer prevention recommendations. The score enables researchers to assess the degree to which people adhere to the Cancer Prevention Recommendations and the health effects of doing so. The WCRF/AICR score is an accumulation of points assigned for adhering – or not adhering – to each of the components of the Cancer Prevention Recommendations or sub-recommendations. However, physical activity won't be included in the triggers.

4.4.4.2 Quality of Life assessments

Quality of Life (QOL) will be assessed pre- and post- intervention (During screening/Week 0 and week 12 +/- 14 days), and 6 months post intervention. Provisionally, we plan on using the European Organisation for Research and Treatment of Cancer Quality of Life Questionnaire Core 30 (EORTC-QLQ-C30)); Quality-adjusted life-year (QALY) (EQ-5D-5L) and a measure of self-care agency/power to self-care (ASAS-R). These will be refined during the Scoping review to limit the number of questionnaires and ascertain the most pragmatic, reproducible and accurate assessment of QOL in this population. We seek to determine the effectiveness of the intervention on Quality of Life (core quality of life of cancer patients and disease). The Quality of Life assessments will provisionally (pending Scoping Review) include the following instruments:

A) The European Organisation for Research and Treatment of Cancer EORTC QLQ-C30 comprises 30 items that can be summarized in 15 scales: Physical Functioning (PF), Role Functioning (RF), Social Functioning (SF), Emotional Functioning (EF), Cognitive Functioning (CF), Global QOL (QL), Fatigue (FA), Pain (PA), Nausea/Vomiting (NV), Appetite Loss (AP), Dyspnea (DY), Sleep Disturbances (SL), Diarrhoea (DI), Constipation (CO), and Financial Impact of Disease (FI). The disease specific HRQOL will be collected using EORTC modules for prostate, urothelial, kidney, penile and testicular cancer as required. These modules have undergone all of the EORTC phases of development (1–4), robust international psychometric testing and rigorous translations according to the EORTC standards.

B) QALY is a generic measure of disease burden, including both the quality and the quantity of life lived. It is used in economic evaluation to assess the value of healthcare interventions. For the economic evaluation, HRQoL is assessed using the EQ-5D-5L [15]. The EQ-5D-5L will be administered at T0, T12. The EQ-5D-5L consists of five questions evaluating the following health dimensions: mobility, self-care, usual activities, pain/discomfort, and anxiety/depression. The patients' EQ-5D-5L health states will be transformed into utility scores.

C) Appraisal Self-Care Agency*:* The ASAS-R is used to measure self-care agency. The 15-item tool has three subscales Having power for self-care; Developing power for self-care and Lacking power for self-care [16].

D) Cancer related fatigue score: The EORTC QLQ-FA12 standardised cancer related fatigue score will be used to measure cancer related fatigue at baseline, end of treatment and the 6 month follow up assessment to determine the impact of this type of intervention programme on cancer related fatigue.

4.4.4.3 Qualitative Interviews

Participants may also be invited to partake in an interview to provide feedback after completion of the programme, to aid in service/intervention improvement in future. These interviews will take place in person or utilising UCC MS Teams. These interviews may be recorded, only with the participants consent, to allow for accurate transcription and documentation of the interview. Recordings will be stored on secure UCC internal servers only and deleted as soon as the recording has been transcribed and documented. These interviews will be optional and not mandatory.

4.4.5 Duration of the Programme

Patients will participate in the programme for a total of up to12 months. They will complete surveys during the first 12 weeks of the study (Screening/Baseline and End of Treatment) and then at 6 months post intervention. Qualitative Interviews will take place within the 6 month post-intervention timeframe. In addition, participants will be invited to voluntary PPI engagement meetings quarterly after completion of the 12-week intervention programme for the duration of the study period (2 years).

4.4.6 Electronic Patient Reported Outcomes (ePRO) Enrolment and Procedures

Electronic Patient Reported Outcomes (ePROs) may be used to collect data directly from patients, specifically the quality of life questionnaires and any other self-reported, questionnaire-based tools, using a pseudoanonymised electronic data capture (EDC) system, CASTOR. This service is offered through the Clinical Research Facility-Cork (CRF-C), and meets national and international guidelines with respect to data privacy and protection standards. CRF-C staff will support the development of the ePRO survey template, study database construction and provide investigator training for downstream data collection, monitoring and analyses. Patient names will not be sent to the EDC database.

Patients will be emailed a link to the ePRO Symptom Survey at the specified timepoints (see schedule of activities) during the study period as required. Surveys can be accessed through any standard internet browser. Responses will be immediately uploaded to the study EDC database.

4.4.7 Withdrawal/Discontinuation of Subjects

All patients who initiate the study will be included in the overall analysis. All reasons for discontinuation of participation in the trial will be documented clearly in the trial record.

The reasons for discontinuation may include:

a) At subject’s own request.
Note: The reason for discontinuation from the study must be documented.

b) Death.
c) Intercurrent illness or condition that would, in the judgment of the principal investigator, affect assessment of clinical status to a significant degree.
d) Evidence of disease recurrence during study.
e) Study is terminated for any reason.
f) Subject withdraws consent for follow-up.

4.4.7.1 Replacement of Subjects

Note: If a participant withdraws from the intervention within the first 4 weeks of the 12-week intervention programme or after consent but prior to the 12-week programme commencing, an alternative participant may enroll in the programme, provided they are willing to join at that point of the programme.

4.4.8 Usability and Satisfaction

In order to gather information on usability and satisfaction with the intervention, the opinions of patients will be gathered using a modified five-item Usability and Satisfaction Survey [17,18]. The questions in this index are designed to assess different domains of usability related to the programme, including usefulness, ease of use and satisfaction with use, and were adapted from a previously developed computer usability and satisfaction questionnaire. The survey will also include some open-ended questions allowing participants to share their perspectives more freely.

As part of the process evaluation, all patients, some health care professionals and some of the broader team involved in the development and implementation of the programme will be invited to provide feedback after the completion of their involvement in the study (in person or remotely). Attendees will be asked about their experiences of the survivorship programme and associated processes, limitations, challenges and possible benefits and future possibilities concerning the intervention. A questionnaire with open ended questions will be provided based on the outcome of the rapid review.

Participants in the 12-week programme will also be invited to quarterly PPI engagement sessions to provide group feedback on the programme, and some participants may be invited to partake in the steering group as additional PPI members.

The interview will be audio-recorded with the consent of participants (both patients and HCPs), and will last approximately one hour. Transcribed audio will be analysed using qualitative content analysis. A priori it is anticipated that we will interview up to twenty patients, ten members of the MDT and ten broader team/stakeholders/service providers. A lesser number may be required if data saturation is attained earlier.

4.4.9 Follow up

Follow up for routine medical oncology care and surveillance will take place as normal, through the medical oncology clinics, see Figure 1. Flowchart. Study specific follow up will take place 6 months after completion of the 12-week intervention programme.

4.4.10 Data extracted from patient hospital records

Patient data including age, sex, cancer site, diagnosis, treatment, current medications will be extracted from the patient records exclusive of patient identifiers from The Orchid Centre database located in Cork University Hospital. All survivorship services that were provided by the CUH and used during the study period will be extracted from the patient records and from patient reports.

5.0 Schedule of Activities

Schedule of Activities. Assessments to be performed at the times stipulated in the table and as clinically required in the management of the patient.

**Timepoint**	Screening (-6 weeks)	Week 1	Week 2	Week 3	Week 4	Week 5	Week 6	Week 7	Week 8	Week 9	Week 10	Week 11	Week 12	EoT	Follow-up (6 Months)
Phone call and patient information leaflet	X														
Written informed consent	X														
Demographics	X														
Previous treatments for cancer	X														
Medical and surgical history	X														
Formal verification of eligibility criteria	X														
Vital signs, including weight	X													X	
Physical exam	X													X	
ECOG performance status	X													X	
Current medication List	X														
Baseline physical assessment	X														
Body composition assessment[1]	X													X	

Muscle strength and mass assessment[2]	X													X	
Physical function and cardiovascular fitness assessment[3]	X													X	
WCRF/AICR Questionnaire	X													X	
**Timepoint**	Screening (-6 weeks)	Week 1	Week 2	Week 3	Week 4	Week 5	Week 6	Week 7	Week 8	Week 9	Week 10	Week 11	Week 12	EoT	Follow-up (6 Months)
Baseline comprehensive nutrition assessment/ education[4]	X														
MST Score Assessment	X													X	
24-hour Dietary Recall	X													X	
Food Frequency Questionnaire	X														X
Programme Induction		X													
Nursing Assessment		X		A		A		A		A		A	X	X	X
EORTC- QLQ-C30 Questionnaire	X													X	X
ASAS-R Questionnaire	X													X	X
EORTC Cancer Related Fatigue Score	X													X	X

Signposting to Other Services[5]	X	X	X	X	X	X	X	X	X	X	X	X	X	X	X
Social Work Education[6]				X											
Nutritional Assessment/Education[7]			x		X		x		X		X				
Occupational Therapy/physiotherapy[8]						X									
Nurse led education[9]								X							
Pastoral care (spirituality & emotional reasoning)										X					
Usability, Feasibility & Satisfaction Feedback questionnaires[10]														X	
PPI workshop invitation and Invitation to Qualitative Interview[11]														X	
Qualitative Interview (not all participants)														X	X

Abbreviations:

X – Scheduled Activity

A – Available on request

EoT – End of Treatment (**Note**: EoT assessments to be carried out during week 12 of intervention programme or within 2 weeks of completing the programme).

Footnotes:

[1] Body composition assessment to include Bioelectrical Impedance Analysis [BIA]/ultrasound assessment

[2] Muscle strength and mass assessment to include 8 repetition maximum, thigh circumference

[3] Physical function and cardiovascular fitness assessment to include grip strength, 6-minute walk test [6MWT]

[4] Comprehensive nutrition assessment and education to include comprehensive diet history, anthropometric measurements, dietary analysis & dietary advice

[5] Services include: Psychosocial Coaching, ARC House, Pharmacist Medication Review, Nursing Review, Medical Review, Social Work Supports, Psycho-Oncology Supports, Other – as required. **Note**: Referral to these services will be an available option throughout the programme, with particular signposting and reminder sessions as highlighted in the schedule of activities.

[6] Social Work education session should include stress management and psychosocial support

[7] Nutritional assessment and education is aimed to revisit dietary analysis results and adjust tailored advice as appropriate

[8] Occupational Therapy/Physiotherapy should include fatigue management, pacing strategies and sleep hygiene.

[9] Nurse led education session on symptom management of side effects of treatment.

[10] Patient feedback questionnaires and thank you session.

[11] Invitation to quarterly PPI engagement meetings and PPI workshops to provide feedback for service improvement. Invitation to qualitative interviews serves to provide feedback and aid programme improvement.

6.0 Statistical Plan

6.1 Sample size justification

Parallel Sampling Groups will be used from Q3 of Year 1 on (after completion of the 6 participant Initial Phase). A new group of 6 participants will commence the programme every 6 weeks, in order to facilitate workload and resource management (see Figure 2, Section 3.2.1). This will mean two groups are at two different timepoints in the programme at any given date. 12 groups of 6 men each are planned in total, including the pilot phase group, with a total anticipated accrual of 72 participants over two years.

Given that the main objectives of this study are to evaluate the *feasibility* of introducing a men's malignancy survivorship programme, we have not calculated the sample size that would be required to reliably estimate the effect of the programme on patient outcomes. This follows from established best practices in pilot and feasibility studies [19] for which there is usually too much uncertainty in the various factors that are needed to make a sample size calculation robust (e.g. effect sizes of interest, outcome variability, etc.). Our sample size justification is instead based on trying to recruit the largest possible sample given existing financial and pragmatic constraints. This will allow us to maximize our ability to identify barriers to implementation, as well as the information that will be required to properly design any subsequent trials of the programme's efficacy (e.g. robust estimation of estimator variability, even for binary outcomes [20]). At the same time, the potential costs of over-recruitment are largely absent for this study. Most importantly, there are no excess risks to patients receiving the intervention, and all patients are guaranteed access to the current standard of care. Further, most of the financial costs of the study are fixed, regardless of the actual number of patients recruited into the study.

Given that the goal of a feasibility study is to identify problems that would impede the conduct of a larger, efficacy study, we have set the sample size at 60 based on advice from Viechtbauer et al. [21], which is aimed at being able to detect failures in study processes that would occur just 5% of the time (with 95% confidence). A sample size of 72 allows for sample attrition from men withdrawing, illness, or waning commitment as commented.

6.2 Statistical Analysis Plan:

Quantitative data will be analysed using SPSS and presented using percentages, means (SD), modes, medians (IQR) as appropriate.

Qualitative data interviews will be digitally recorded, verbatim transcripts will be prepared from the sound files, transcripts checked for accuracy against the sound files and anonymised. Thematic content analysis will be used to code data in transcripts that is relevant to the process evaluation. NVivo software will be used to assist with the analysis. One researcher trained in qualitative research methods will analyse the qualitative data allowing for fuller immersion and to obtain an overall sense of the data. Initial open coding will be organised into higher level coding, and

thematic interpretations. Data analysis will be iterative such that early interviews can inform questions in later interviews. Quantitative and qualitative data will be integrated to provide an overall perspective on the process of implementing the intervention/study. We will do that by describing what was delivered in the intervention, what process effects were observed, then identify explanatory 'Context + Mechanism → Process effect' alignments that explain how the intervention, and the study more broadly, was perceived by participants, if/why this varied, and how these perceptions affected receptivity to the intervention.

Once data entry is finalized, study data will be assessed for incompatible, discrepant or clinically implausible values. Outlying values for all distributions, in isolation and over time, will be identified. Any concerning data will be reconciled against original source data. Following completion of cleaning the database will be locked.

The study sample will be described in detail. Continuous variables will be described by their means and SDs, medians and IQRs, and their range; while categorical variables will be described by their counts and percentages in each category.

Feasibility outcomes will be similarly described. These include the number of enrolled patients who complete the baseline and follow-up assessments, and the number of patients who partake in all planned activities versus less than all; the number of patients that require medical review and the timeframe to Medical review; changes in muscle strength and mass from baseline to end of programme assessments; changes in dietetic assessments from baseline to end of programme assessments; changes in QOL outcome measures from baseline to end of programme assessments; the number of patients enrolled in the programme; extra Health Care Professional time required and resources required for the intervention; and reasons for not completing the programme will be collected through Drop Out Forms and with qualitative discussions with HCP involved in the programme.

Missing data will be evaluated, and based on what we observe, dealt with in whatever manner we find appropriate based on current best practices. All analyses will be conducted using the R Project for Statistical Computing and the RStudio IDE. All trial reporting will be done following CONSORT and the CONSORT addendum for pilot/feasibility trials [22].

All analyses will be conducted and/or supervised by the HRB CRF-C Principal Statistician (Dr Darren Dahly), under their established quality systems and SOPs, and in accordance with *ICH E9 Statistical Principles for Clinical Trials* and *ICH E6 Good Clinical Practices.*

7 .0 Safety and Adverse Events

7.1 Recording of Adverse Events

Adverse events (AE) of interest (those deemed potentially related to the intervention) will be recorded and assessed for relationship to the intervention programme throughout the study period. Adverse Events will be documented and graded in accordance with Common Terminology Criteria for Adverse Events (CTCAE) v5.0. Patient reporting of an adverse event to an HCP will trigger recording of the AE, these will not be assessed routinely at every visit due to resources.

Serious Adverse Events (SAE's), defined for the purposes of this study as CTCAE v5.0 grade ≥3 adverse events of any kind, or those grade <3 but deemed by an investigator to be serious, will be reported to the LIAM Mc Steering Group for review at the following meeting. SAE's of grade ≥4 will trigger a pause in the study for safety reasons until the AE has been formally assessed by the Steering Group and a decision made to either proceed with or terminate the study.

We do not anticipate any adverse events associated with completion of the patient reported outcomes. Patients will have the option of skipping questions they do not wish to answer. In event that study participation does result in any event that has significant negative consequences for the subject, this will be recorded by an investigator in the EDC (Electronic Data Capture) and will be reported to the Sponsor and Ethics committee.

8.0 Data Handling and Record Keeping

8.1 Confidentiality

Pseudoanonymised study data will be maintained in a dedicated CASTOR database in a secure password-protected location on the UCC server network. A unique study ID number will be assigned to each subject. This will be linked to the patient identifying information by a list maintained by the PI in a secure location, separate to other study documentation. This is primarily to facilitate subsequent follow-up of patients. The patient identity list will remain at the clinical site and will not be forwarded to any third party, including the EDC platform. The data will be transported by the delegated team member from the Mardyke Arena to the clinical site (CUH). It will be available for review by the study monitor, auditors or inspectors as required. University College Cork, as study Sponsor, is the data controller for the research database.

8.2 Source Documentation

Electronic and/or paper case report form (CRF) will be used as source documentation in this project.

8.3 Case Report Forms

Pseudoanonymised data will be entered onto study specific eCRFs. With the EDC platform, almost all actions performed by users are tracked. It is possible to review what each user did in any field. Any missing data will be explained. If a data point cannot be answered due to missing data or other known reason, the EDC platform allows for this to be noted, any item on the CRF left blank because the procedure was not performed will be denoted by “N/D”. Items not applicable to the individual case, will be recorded as “N/A”. All entries should be printed legibly in black ink where paper used. In correcting any error, a single straight line will be drawn through the incorrect entry without obscuring the original entry and the correct entry recorded above it. All such changes must be initialed and dated as per ICH-Good Clinical Practice (GCP).

8.4 Records Retention

Study documents and pseudoanonymised data will be kept for 10 years as per UCC Code of Research Conduct (Data Protection Notice v4.1) after the end of the study. Thereafter, the data may be stored for a further period of time for legal reasons (e.g. revised retention obligations), or more if required by law. Study documents will thereafter either be destroyed or fully anonymized and retained, as scientific interests dictate at that time, in line with UCC’s Data Protection Policy and the UCC Code of Research Conduct.

9.0 Ethical Considerations

This study will be conducted in accordance with the Declaration of Helsinki, the applicable sections of ICH GCP, and the terms of approval of the responsible Ethics Committee, the Clinical Research Ethics Committee (CREC) of Cork Teaching Hospitals. This study was submitted for Full ethical approval by the CREC on October 14th 2022. If any data breach is detected during the study, it will be reported in <72 hours to the Data Protection Commission (DPC).

10.0 Study Finances

10.1 Funding Source:

The costs of this study will be funded by an Irish Cancer Society grant (MHI22BAM) for 2 years for a total of 299,988€ including personnel and non-personnel costs. Additional part-funding for some data analysis and management is available through the HRB UCC Cancer Trials Group (CTIC 2021-02) grant.

Patients will not be paid for taking part in the study. Patients travel costs will be covered to ensure they are not left out of pocket.

This patient access fund exists to ensure that no man is denied access to the programme or to be involved as a PPI representative for want of travel costs, interpreter costs, or other unforeseen costs. This aims to ensure that men from underserved communities, at geographic distance from the CUH, and those with an unmet need can participate fully in this programme.

10.2 Indemnity for the performance of the study

Protocol and prototype indemnity will be provided by UCC. Malpractice indemnity will be provided to research staff as part of Clinical Indemnity Scheme coverage provided by the States Claims Agency.

11.0 Sponsorship

The study is sponsored by University College Cork (UCC).

12.0 References

1. Government of Ireland (2017) National Cancer Strategy 2017-2026. Government of Ireland: Dublin. Available at: http://health.gov.ie/wp-content/uploads/2017/07/National-Cancer-Strategy-2017-2026.pdf
2. The Irish Cancer Society Women's Health Initiative: https://www.cancer.ie/cancer-research/about-our-cancerresearch/our-cancer-research-networks/womens-health-initiative
3. The Irish Cancer Society Strategy 2020-2025: https://www.cancer.ie/about-us/irish-cancer-society-strategy2020-2025
4. Bowie J, Brunckhorst O, Stewart R, Dasgupta P, Ahmed K. Body image, self-esteem, and sense of masculinity in patients with prostate cancer: a qualitative meta-synthesis. J Cancer Surviv. 2022 Feb;16(1):95-110. doi: 10.1007/s11764-021-01007-9. Epub 2021 May 8. PMID: 33963973; PMCID: PMC8881246.
5. Greenhalgh T, Wong G, Jagosh J, et al. Protocol—the RAMESES II study: developing guidance and reporting standards for realist evaluation. BMJ Open 2015;5:e008567. doi: 10.1136/bmjopen-2015-008567
6. Moore GF, Audrey S, Barker M, Bond L, Bonell C, Hardeman W, Moore L' O'Cathain A, Tinati T, Wight D, Baird J. Process evaluation of complex interventions: Medical Research Council guidance. BMJ. 2015 Mar 19;350:h1258. doi: 10.1136/bmj.h1258. PMID: 25791983; PMCID: PMC4366184.
7. Saab, M.M., Landers, M., Cooke, E. et al. Feasibility and usability of a virtual reality intervention to enhance men's awareness of testicular disorders (E-MAT). Virtual Reality 23, 169–178 (2019). https://doi.org/10.1007/s10055-018-0368-x
8. Shams-White MM, Brockton NT, Mitrou P, Romaguera D, Brown S, Bender A, Kahle LL, Reedy J (2019). Operationalizing the 2018 World Cancer Research Fund/American Institute for Cancer Research (WCRF/AICR) Cancer Prevention Recommendations: A Standardized Scoring System. Nutrients, Jul;12;11(7):1572.
9. Baumgartner, F. R., & Walker, J. L. (1988). Survey Research and Membership in Voluntary Associations. American Journal of Political Science, 32(4), 908–928. https://doi.org/10.2307/2111194
10. Chamney PW, Wabel P, Moissl UM, Müller MJ, Bosy-Westphal A, Korth O, Fuller NJ (2007) A whole-body model to distinguish excess fluid from the hydration of major body tissues. *Am J Clin Nutr*. Jan;85(1):80-9.
11. Fess EE (1986). The need for reliability and validity in hand assessment instruments. *J Hand Surg Am*, 11(5).
12. Welch AA, Luben R, Khaw KT, Bingham SA. The CAFE computer program for nutritional analysis of the EPIC-Norfolk food frequency questionnaire and identification of extreme nutrient values. J Hum Nutr Diet. 2005 Apr;18(2):99-116. doi: 10.1111/j.1365-277X.2005.00593.x. PMID: 15788019.
13. Ferguson M, Capra S, Bauer J, Banks M. Development of a valid and reliable malnutrition screening tool for adult acute hospital patients. Nutrition. 1999 Jun;15(6):458-64
14. Buchholz I, Janssen MF, Kohlmann T, Feng YS (2018). A systematic review of studies comparing the measurement properties of the three-level and five-level versions of the EQ-5D. Pharmacoeconomics 36(6):645-661.

15. Danquah, I.H., Petersen, C.B., Skov, S.S. et al. Validation of the NPAQ-short – a brief questionnaire to monitor physical activity and compliance with the WHO recommendations. BMC Public Health 18, 601 (2018). https://doi.org/10.1186/s12889-018-5538-y
16. Sousa, V. D., Zauszniewski, J. A., Bergquist-Beringer, S., Musil, C. M., Neese, J. B., & Jaber, A. A. F. (2010). Reliability, validity and factor structure of the Appraisal of Self-Care Agency Scale–Revised (ASAS-R). Journal of evaluation in clinical practice, 16(6), 1031-1040.
17. Lewis, J. R. (1995). IBM computer usability satisfaction questionnaires: Psychometric evaluation and instructions for use. International Journal of Human–Computer Interaction, 7(1), 57–78.
18. Sharma, P., Dunn, R. L., Wei, J. T., Montie, J. E., & Gilbert, S. M. (2016). Evaluation of point-of-care PRO assessment in clinic settings: integration, parallel-forms reliability, and patient acceptability of electronic QOL measures during clinic visits. Quality of Life Research, 25(3), 575-583.
19. Whitehead, A. L., Sully, B. G., & Campbell, M. J. (2014). Pilot and feasibility studies: is there a difference from each other and from a randomised controlled trial? Contemporary clinical trials, 38(1), 130-133. DOI:https://doi.org/10.1016/j.cct.2014.04.001
20. Teare, M. D., Dimairo, M., Shephard, N., Hayman, A., Whitehead, A., & Walters, S. J. (2014). Sample size requirements to estimate key design parameters from external pilot randomised controlled trials: a simulation study. Trials, 15(1), 264 https://trialsjournal.biomedcentral.com/articles/10.1186/1745-6215-15-264
21. Viechtbauer, W., Smits, L., Kotz, D., Budé, L., Spigt, M., Serroyen, J., & Crutzen, R. (2015). A simple formula for the calculation of sample size in pilot studies. Journal of clinical epidemiology, 68(11), 1375-1379. PMID: 26146089; DOI: 10.1016/j.jclinepi.2015.04.014
22. Eldridge SM, Chan CL, Campbell MJ, Bond CM, Hopewell S, Thabane L, Lancaster GA; PAFS consensus group. CONSORT 2010 statement: extension to randomised pilot and feasibility trials. BMJ. 2016 Oct 24;355:i5239. doi: 10.1136/bmj.i5239. PMID: 27777223; PMCID: PMC5076380.

13.0 Study Documents Versions Log

IMPORTANT NOTE: The following documents have their own version and date and have been ethics approved as individual documents.

Study Title: The LIAM Mc Trial - Linking In with Advice and supports for Men impacted by Metastatic cancer

Short Title: The LIAM Mc Trial **Study Reference:** **Sponsor:** University College Cork (UCC) **UCC code:** 22052

CREC REFERENCE:	First ethics approval<br>Sent:<br>Approval Date: CREC rev ref:	Amendment #1	Amendment #2	Amendment #3	Amendment #4	Amendment #5
Protocol	24-Nov-22	Physio CRF, amend definition of exercises and time taken. Added Bi-weekly data collection	Change to Dietician CRF,	Addition to Dietician CRF, individual patient assessment document and data collection	Nurse CRF and CNS CRF change due to change of PI name	
