# Supplementary material for: Study protocol: LIAM Mc trial (Linking In with Advice and supports for Men impacted by Metastatic cancer)
Source: PLoS One. 2025 Apr 3;20(4):e0313454. doi: 10.1371/journal.pone.0313454 (PMC11967965; doi:10.1371/journal.pone.0313454)
Supplement: S3 File — (PDF) [file pone.0313454.s003.pdf]

**Weeks** *(Twice weekly sessions)*	**Exercise**	**Sets & Reps**	**RPE**	**Resistance**	**Purpose**
**1-3**	BC, LE, HC	3*8	4-6	Moderate	Hypertrophy and Endurance
	TE, STCR, SCR	3*12			
**4-6**	BC, LE, HC	3*7	5-7	Heavy	Strength not maximal but highly demanding
	TE, STCR, SCR	3*12			
**6-9**	BC, LE, HC	3*6	6-8	Heavy	Strength
	TE, STCR, SCR	3*12			
**9-12**	BC, LE, HC	3*5	8+	Very heavy	Strength
	TE, STCR, SCR	3*12			

Supplementary Figure 1. Sample Exercise Programme: 12 week progressive resistance plan

BC, bicep curl; LE, leg extension; HC, hamstring curl; TE, tricep extension; STCR, standing calf raise; SCR, seated calf raise; RPE, Rate of perceived exertion.

Week	Aerobic exercise (Bike)	RPE	Recovery(minutes)
1	3*30 secs	4-6	2
2	4*30	4-6	2
3	5*30	4-6	2
4	6*40	5-7	2
5	6*40	5-7	2
6	6*40	5-7	2
7	6*50	6-8	2
8	6*50	6-8	2
9	6*50	6-8	2
10	6*60	8+	2
11	6*60	8+	2
12	6*60	8+	2

Supplementary Figure 2. Sample Exercise Programme: 12-week progressive Aerobic exercise plan

RPE, Rate of perceived exertion.
